# Oral Surgery

**Published:** 1909-05-15

**Authors:** Wm. E. Chenery

**Affiliations:** Boston, Mass.


### ORAL SURGERY.

BY DR. WM. E. CHENERY, BOSTON, MASS.

Of late years much attention has been given to mouth-breathing. Its effects have been studied not only by the rhinologist but by the dentist, and it has been justly found to be the wide-open door to many diseases.

To the dentist, persistent mouth-breathing means in adolescence deformed dental arch and irregular teeth, the opportunity of harboring multitudes of bacteria favoring the people's disease—dental caries—and inviting tuberculosis and gastric disorders.

The rhinologist thinks of the perverted air-current and its bad effects. The inspired air in passing through the normal nose is strained from dust and bacteria, is warmed and moistened, and after having been thus purified, is prepared for entering the trachea and lungs without causing irritation. This is very imperfectly done when the air passes through the mouth. Persistent mouth-breathing causes dulled sense of smell and taste, interference with hearing and often deafness, changes in the quality of the voice, and frequently catarrhal conditions. Lack of drainage and ventilation of the nose also means increased irritation and opportunity for infection. There is also interference with proper oxygenation. In fact, good health and facial beauty demand a normal nose rightly used at all times. The most frequent causes of mouth-breathing are as follows: (1) adenoids, (2) enlarged tonsils (3) deviated septa, (4) hypertrophied turbinates, and (5) polypi; and we should always remember habit as a cause, if these obstructions have been removed. I believe all dentists should have a good working knowledge of these conditions, and a closer relationship between dentist and rhinologist is desirable. To-day we shall consider adenoids, and to-morrow the other subjects referred to.

In my clinic at the Boston Dispensary I have recently examined the records of 3,000 children under fifteen, and I find the book diagnosis entered in nearly sixty-five per cent. to be adenoids or adenoids and enlarged tonsils. Ballenger says it has been estimated that adenoids are present in children otherwise normal in from one to nine per cent. of the cases examined. Sill says that twenty to twenty-five per cent. of children in his general clinic have adenoids or have had them removed. In deaf-mutes the percentage is much larger—fifty to seventy-five per cent. Seventy-five per cent. of ear troubles are due to adenoids. Kyle says that ninety per cent. of patients having adenoids have some degree of deafness. Woakes says ninety-five per cent. Dench that more than half show trouble with the ear. In a series of 120 cases, twenty-six had lung troubles.

It is estimated that eighty-four per cent. of chronic nasal diseases are due to adenoids. According to Wells, adenoids constitute eighty-eight per cent. of all affections of the vault of the pharynx and twenty-five per cent. of all diseases of the upper throat, generally including the fauces. Faught states that the high contracted arch and respiratory obstruction are coincident in about forty-three per cent. of the cases. I feel sure that if a more careful examination were made this would be found in nearly all the cases.

The cause of respiratory obstruction which leads to mouth breathing in children and to irregular dentition is preeminently adenoids.

In 1876, Joseph Meyer, of Copenhagen, first described this condition, and to him the world owes much for improved health and longevity.

Holt says, speaking of adenoids: "It is a very common condition and one very much neglected by the general practitioner. It is the source of more discomfort and the origin of more minor ailments than almost any other pathological condition in children.

Adenoids are hypertrophied lymph glands situated at the upper and posterior part of the naso-pharynx or, as we often say, the vault of the pharynx. Lymphoid tissue should normally exist in the post-nasal space, but by irritation, inflammation, and infection this velvety tissue becomes chronically enlarged and so materially obstructs the normal air-current. The smaller the naso-pharynx the more it obstructs if hypertrophied. At the sides of the vault are the internal openings of the Eustachian tubes, and just posterior the foss*æ* of Rosenmuller are situated. Adenoids often extend into this space and by closer relationship become a great source of danger to the Eustachian tubes and the ears. Heredity and climate seem to have little effect as a cure for adenoids. The family nose may be a factor.. Sex makes no difference. Scheppegrell reports six and one-half per cent. of negroes in the total cases of adenoids in his clinic. The negro has broad, open nostrils, and therefore he has less actual obstruction from adenoids.

The presence of adenoids can often be traced to an attack of influenza, scarlet fever, measles, or some other of the children's diseases. Infection of the lymphoid and epithelial tissue is more apt to occur in childhood, for it is soft and friable and therefore susceptible. Adenoids may be found soon after birth. The period in which they are most frequently found is from three to twelve years. There is a tendency for atrophy to occur in adolescence at about fifteen, but this is by no means to be depended upon, as I have often operated on adults, and recently on a man of forty-two with large, soft adenoids.

SYMPTOMS.

We may have snuffles, frequent head colds, requiring mouth-breathing, which is usually noisy; sleep is restless, often night terrors and enuresis are associated. With development we find the narrow, pinched face and slit-like nostril, the *alæ* collapsing from non-use; the lips are parted, the upper lip becomes foreshortened and the upper central incisors are shown with a tendency to crowd together or overlap with the V-shaped instead of the dome-shaped arch, and consequently with dental irregularity. The bridge of the nose thickens and there is a general listless, stupid expression to the face. Earache and dullness of hearing are frequent. Aprosexia or inabitily to learn or fix the attention is common. The child is apt to be pale, under-developed, narrow-chested, or with actual chicken-breast. Epilepsy and chorea are often brought on by the presence of adenoids.

Clinical observation shows that if a child is going to have adenoids he will have them before eight years of age; between five and eight is the most prevalent period, and this is the time when they do the most harm in disturbing the second dentition.

There is no use in temporizing with medicine or waiting, because there is a tendency to atrophy at the age of fifteen. Irreparable harm may be done, and certainly nothing is gained. Operation is advisable in all cases where adenoids cause interference with nasal respiration, and especially if the ears are affected. An operation should be made early, before eight years. Occasionally the adenoids recur, but if a thorough operation is performed, and the sides of the *fossæ* of Rosenmuller are thoroughly curetted, recurrence is rare. Large tonsils should be removed at the same operation, usually before the adenoids.

There is a tendency to refer cases of irregular teeth to the orthodontist. The busy practitioner has no time for orthodontia. This is all right when the irregularities are pronounced and need much attention, but this is an age of preventive medicine, and whenever irregularities is due to mouth-breathing, it can and should be avoided. Parents are learning the necessity of caring for children's teeth early, and it is the dentist more than the family physician who is responsible for the form of the mouth and face, and for the proper eruption of the teeth during the plastic stage—seven to fourteen—when the bony framework of the face is easily moulded. In the deciduous dentition irregular teeth are rarely found, and the first permanent molars are generally erupted regularly, but with the second dentition the trouble generally begins. The first molars should be preserved as long as possible. The dentist should see the patient early, and should detect the presence of that which will cause trouble in a few years, and advise operation. If any obstruction to nasal breathing is removed early, and the mouth-breathing habit is corrected, the superior maxillary bones will develope as they should, the arch will be dome-shaped, the teeth regular, and the occlusion usually perfect. Thumb-sucking and rubber nipples should never be allowed.

Remember the time for operating in order to avoid any deformity is during the plastic stage of the bones of the face. Mouth-breathing becomes almost a necessity even though the obstruction may be removed after the facial bones are molded rigidly into the high V-shaped arch. The danger from the operation in the hands of a skilled operator is very small when compared with the improvement in health. Ether is by far the safest anesthetic. The position for operating depends on the operator.

Finally, let me lay down this rule: Any condition in the nose or upper throat which interferes with the proper passage of air through the nose is a menace to good health. Ventilation and drainage of the upper air passages must be maintained at all times, A patient is never too young for correction of bad habits, but often too old. Bad habits mean mouth-breathing, which is the cause of the vast majority of dental irregularities. By early removing the adenoid or nasal obstruction—before six or eight—making possible the correction of the mouth-breathing habit, the need of orthodontia will be largely avoided, and the work will be better and more easily done. The health of the patient will be improved, his power of resistance will be increased, and his life will be prolonged.—*Dental Cosmos.*

